# Are mitophagy enhancers therapeutic targets for Alzheimer’s disease?

**DOI:** 10.1016/j.biopha.2022.112918

**Published:** 2022-04-04

**Authors:** Jangampalli Adi Pradeepkiran, Ashly Hindle, Sudhir Kshirsagar, P. Hemachandra Reddy

**Affiliations:** a Department of Internal Medicine, Texas Tech University Health Sciences Center, Lubbock, TX 79430, USA; b Department of Pharmacology and Neuroscience, Texas Tech University Health Sciences Center, Lubbock, TX 79430, USA; c Department of Neurology, Texas Tech University Health Sciences Center, Lubbock, TX 79430, USA; d Department of Public Health, Graduate School of Biomedical Sciences, Texas Tech University Health Sciences Center, Lubbock, TX 79430, USA; e Department of Speech, Language, and Hearing Sciences, Texas Tech University Health Sciences Center, Lubbock, TX 79430, USA

**Keywords:** Alzheimer’s disease, Aβ, Phosphorylated tau, Mitochondrial dysfunction, Mitophagy

## Abstract

Healthy mitochondria are essential for functional bioenergetics, calcium signaling, and balanced redox homeostasis. Dysfunctional mitochondria are a central aspect of aging and neurodegenerative diseases such as Alzheimer’s disease (AD). The formation and accumulation of amyloid beta (Aβ) and hyperphosphorylated tau (P-tau) play large roles in the cellular changes seen in AD, including mitochondrial dysfunction, synaptic damage, neuronal loss, and defective mitophagy. Mitophagy is the cellular process whereby damaged mitochondria are selectively removed, and it plays an important role in mitochondrial quality control. Dysfunctional mitochondria are associated with increased reactive oxygen species and increased levels of Aβ, P-tau and Drp1, which together trigger mitophagy and autophagy. Impaired mitophagy causes the progressive accumulation of defective organelles and damaged mitochondria, and it has been hypothesized that the restoration of mitophagy may offer therapeutic benefits to AD patients. This review highlights the challenges of pharmacologically inducing mitophagy through two different signaling cascades: 1) The PINK1/parkin-dependent pathway and 2) the PINK1/parkin-independent pathway, with an emphasis on abnormal mitochondrial interactions with Aβ and P-Tau, which alter mitophagy in an age-dependent manner. This article also summarizes recent studies on the effects of mitophagy enhancers, including urolithin A, NAD^+^, actinonin, and tomatidine, on mutant APP/Aβ and mutant Tau. Findings from our lab have revealed that mitophagy enhancers can suppress APP/Aβ-induced and mutant Tau-induced mitochondrial and synaptic dysfunctions in mouse and cell line models of AD. Finally, we discuss the mechanisms underlying the beneficial health effects of mitophagy enhancers like urolithin A, NAD^+^, resveratrol and spermidine in AD.

## 1. Introduction

Alzheimer’s disease (AD) is the leading cause of dementia in the aging population, and it affects over 55 million people worldwide [1]. The World Alzheimer Report 2019 states that this number will escalate rapidly to 88 million by 2050 [2]. With increasing longevity, AD is becoming an increasingly urgent health concern for society. AD is a progressive neurodegenerative disease that leads to the irreversible loss of neurons and intellectual abilities and eventually causes death within a few years [3]. Currently there are no medical interventions capable of curing or delaying the progression of AD in patients.

The formation and accumulation of beta-amyloid (Aβ) and phosphorylated tau (P-tau) are the two major pathological changes in the brains of patients with AD [4–6]. Aβ is generated predominantly in forms containing 40 and 42 amino acids from amyloid precursor protein (APP) following sequential cleavages by β- and γ- secretases [7,8]. Indeed, Aβ is a neurotoxic peptide and its accumulation has been extensively reported in the development, progression and pathogenesis of AD. Hyperphosphorylation of tau, and its formation of neurofibrillary tangles, is another pathological hallmark and definitive diagnostic feature of AD. The neurons of AD patients undergo sequential functional modifications including inflammation, impaired energy metabolism, overwhelming oxidative stress, and synaptic dysfunction, leading to impaired axonal transport and neuronal death [9–12].

Many risk factors (especially diabetes, obesity, smoking and hypertension) are also associated with an increased risk of cognitive decline with age [13–18]. Like AD, these risk factors contribute to excessive oxidative stress, mitochondrial fragmentation and impaired autophagy and mitophagy.

The purpose of this article is to summarize the recent preclinical efforts to develop mitophagy enhancers as therapeutic drugs for AD. This article also discusses the mechanisms of mitophagy enhancers, particularly via the PINK1-Parkin dependent and independent pathways, in clearing dead and/or dying mitochondria from healthy and diseased neurons.

### 1.1. Mitochondria

Mitochondria are the powerhouses of the cell, which are responsible for ATP production, energy conversion, respiration and other important cellular events including proliferation, redox homeostasis, and apoptosis [19–22]. Mitochondrial function is maintained by balanced mitochondrial dynamics (consisting of mitochondrial fission and fusion) and biogenesis [23,24]. It is important to maintain mitochondrial dynamics and biogenesis for neuronal health. In many age-related neurocognitive disorders, including Alzheimer’s disease and Parkinson’s disease, the dysfunction of mitochondria is correlated with structural damage and defective dynamics, biogenesis, and mitophagy [25–27]. In AD, impaired mitochondrial dynamics, impaired energy metabolism, and overwhelming oxidative stress lead to the mitochondrial dysfunction observed in neurons [28,29].

Importantly, growing evidence indicates that accumulated Aβ and p-Tau within mitochondria can interact with mitochondrial outer membrane proteins like VDAC1, resulting in mitochondrial defects and playing a major role in AD pathogenesis [30]. Intramitochondrial Aβ also interacts with the electron transport chain (ETC), inducing reactive oxygen species (ROS) formation [31]. Accumulating evidence suggests that mitochondrial dysfunction associates with oxidative stress, aging, inactive lifestyle, and excessive caloric intake that can trigger amyloidogenic and ROS-mediated neuroinflammatory processes [32–37].

Mitophagy is a specialized form of autophagy that regulates the elimination of defective mitochondria. Programmed mitophagy plays a pivotal role in the natural turnover of mitochondria. It is regulated by protein machinery that engulfs damaged/aged mitochondria in an autophagosome, which is then fused with a lysosome to initiate degradation [38,39]. The age-dependent decline of mitophagy hampers the elimination of dysfunctional or damaged mitochondria and alters mitochondrial biogenesis [40–42]. Defective mitophagy is correlated with a series of pathophysiological events, such as Aβ- and P-tau-induced toxicities and mitochondrial defects. Aβ, P-tau and Drp1 interactions with PINK1 and Parkin cytosolic proteins, mitochondrial ROS, impaired biogenesis, and other factors are associated with unsuccessful mitophagy in age-related neurodegenerative diseases [43].

### 1.2. Mitochondrial abnormalities

Mitochondria are the centers of the signaling pathways which produce ATP, regulate calcium, and maintain redox homeostasis in healthy cells. Impaired mitochondrial dynamics results in excessive production of ROS, which in turn damages mitochondrial DNA (mtDNA) and ultimately causes defects in biogenesis, mitophagy, and apoptosis.

Mitochondria are dynamic organelles that fuse and divide to form constantly changing tubular networks in most eukaryotic cells. Furthermore, regulation of mitochondrial dynamics is crucial for the health of the cell [44–48]. At any point in the cell cycle, a mitochondrion may undergo fission to give rise to two separate mitochondria [49]. Simultaneously, mitochondria are undergoing fusion in which both the inner and outer membranes of the mitochondria break and rejoin to form a single intact mitochondrion. The mitochondrial fission and fusion processes maintain a dynamic reticular network that spreads throughout the cytosolic volume and responds to extrinsic and intrinsic pathological stress signals.

Mitochondria are dynamic organelles, and their shape is controlled by a balance of fusion and fission events, which are governed by the proteolysis and posttranslational modification of several proteins [50–52]. The known proteins that are involved in mitochondrial fission are Drp1 and Fis1, and mitochondrial fusion proteins include MFN1, MFN2, and OPA1 [53–56]. In a healthy state, Drp1 is important for mitochondrial division, distribution, and synaptic functions; In AD, abnormal interactions of Drp1 with Aβ and P-tau enhance the GTPase activity of Drp1, increasing mitochondrial fragmentation, impairing healthy mitochondrial dynamics, and ultimately leading to defective mitophagy and synaptic dysfunction in AD [57]. Aβ, P-tau, and Drp1 also interact with the mitochondrial outer membrane proteins like PINK1 and PARKIN, reducing mitophagy in AD neurons which prevents healthy turnover of old or damaged mitochondria [58,59]. Finally, AD neurons have diminished expression of PGC-1α, Nrf1, Nrf2, and TFAM, impairing the biogenesis of new mitochondria to replace those that have been damaged or removed [60].

## 2. Mitophagy

Mitophagy is a highly complex cellular process that regulates both mitochondrial quality and quantity. It is used to conveniently eliminate mitochondria that have been critically damaged [61–63].

Both morphological and biochemical markers have been described for mitophagy, and the use of a combination of approaches to measure mitophagy is strongly recommended. Despite the significant recent advances in our knowledge of selective mitophagy, more reliable and accurate quantitative assays to monitor mitophagy need to be developed. Interest in mitophagy has increased during recent years, mostly by virtue of the growing awareness that germline mutations in key mitophagy proteins, like PINK1 or Parkin, are frequently observed in patients with neurodegenerative diseases [64,65]. Mitophagy plays a pivotal role in the natural, regulated machinery of the cell cycle and disposes of the mitochondrial waste during mitosis. Mitophagy allows for the orderly degradation and recycling of damaged mitochondria and utilizes unique membrane trafficking processes [66,67]. Maintenance of mitochondrial quality and number is important for normal cellular metabolic homeostasis. Mitophagy promotes mitochondrial quality and prevents the accumulation of dysfunctional mitochondria that can lead to cellular degeneration.

Mitophagy begins with the formation of the phagophore which expands through lipid acquisition to become the autophagosome [68]. Following the selective engulfment of a defective mitochondria, the mitochondria is subsequently catabolized by fusion of the autophagosome to a lysosome to form an autolysosome where the enveloped contents are degraded [69–72]. How the autophagosome formation occurs is still not clear. However, studies have suggested that the plasma membrane, the Golgi complex, the endoplasmic reticulum (ER), and mitochondria are involved in the process [73]. Many studies have reported that autophagosomes that select and degrade defective mitochondria (sometimes called mitophagosomes) are formed by ER-mitochondria interactions in mammalian cells.

Nearly 30–45 autophagy-related genes (ATG genes) are involved in autophagosome formation in yeast. Autophagy involves the recruitment of regulatory protein complexes which include: (1) The ULK kinase complex: ULK1-ATG13-FIP200, (2) the phosphatidylinositol 3-kinase (Ptdlns3K) complex: Beclin1-ATG14-Vsp15-Vsp34-AMBRA1, (3) the ATG9-ATG2-ATG18-ATG3-ATG10 complex and (4) the ATG5-ATG12-ATG16 and ATG7/LC3 conjugation systems. Once assembled, the phagosome is ready to selectively engulf the defective or damaged mitochondria for degradation through mitophagy. Mitophagy is broadly classified into two types: a) PINK1/Parkin-dependent, and b) PINK1/Parkin-independent, as described in the following subsections.

### 2.1. PINK1/Parkin-dependent mitophagy

**Fig. 1.**
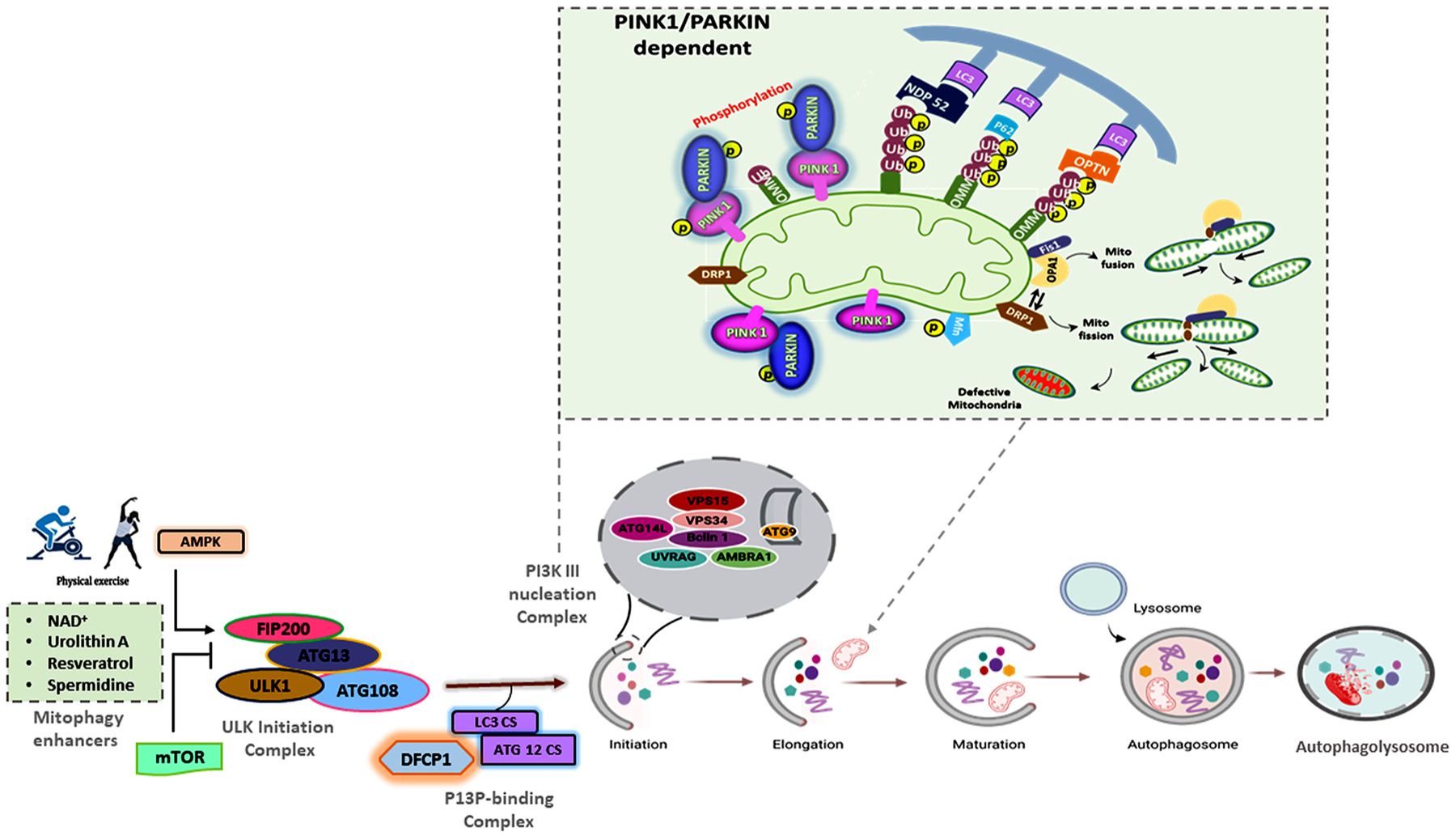
Mechanism of PINK1–Parkin-dependent mitophagy. (A) Mitophagy induction is normally activated by AMPK and inhibited by mTOR. Here, the pharmacological induction of mitophagy by NAD^+^, urolithin A, resveratrol, or spermidine will activate the ULK1 complex (FIP200, ATG13, ULK1 and ATG108) to initiate the P13P-binding complex. The PI3KIII nucleation complex activates VPS34, VPS15 lipid kinase, beclin, UVRAG, and AMBRA1 which participate in these processes, including the stabilization of ULK1 and binding to the PI3KIII complex. PINK1 on the outer mitochondria membrane (OMM) is activated by mitochondrial depolarization and autophosphorylation. Activated PINK1 phosphorylates and recruits Parkin which ubiquitinates several OMM components. Ubiquitin chains attached to the OMM are subsequently phosphorylated by PINK1 serving as a signal for the autophagic machinery. The adapter proteins p62, OPTN, and NDP52 recognize phosphorylated poly-Ub chains on mitochondrial proteins and initiate autophagosome formation through binding with LC3B. Formation of a double-layered membrane phagophore formation within the cytosol requires the PI3KC3 complex. The complex then binds ATG proteins, resulting in the recruitment of lipids to form the phagophore; cytoplasm and organelles are engulfed during the elongation of the phagophore, forming an autophagosome. In the final step of the process, lysosomes fuse with the autophagosome, releasing lysosomal hydrolases into the interior, resulting in degradation of the vesicle contents, including mitochondria.

In PTEN-induced putative kinase 1 (PINK1)/Parkin-dependent mitophagy, mitochondrial ubiquitin chains are recognized by autophagic ubiquitin receptors. Dysfunctional mitochondria tend to have depolarized membranes with reduced proton gradients across their inner mitochondrial membranes (IMM). This depolarization allows PINK1 kinase to accumulate at the outer mitochondrial membrane (OMM) and phosphorylate mitochondrial surface proteins including PINK1, Parkin, Mfn1 and Mfn2. Phosphorylation and ubiquitination by PINK1 of Ser65 of the E3 ubiquitin ligase Parkin initiates the ubiquitination of several substrates in the outer mitochondrial membrane [74]. Phosphorylation of ubiquitin chains leads to the recruitment of several autophagy adapter proteins, which interact, with the ubiquitin chains and LC3, such as the adapter proteins p62, OPTN, and NDP52, which drive mitophagy by binding to a phagophore. LC3 also functions as an adapter protein to recruit selective cargo to the autophagosome via interactions with lysosome cargo receptors. PINK1 and Parkin-mediated mitophagy is triggered by poor mitochondrial quality, which is in turn determined by the cumulative activities of other mitochondrial proteins such as Drp1, Fis1, Mfn1, Mfn2, and PGC1a. Dysfunctional mitochondria fail to maintain their membrane potentials. This activates PINK1, which subsequently leads to Parkin recruitment, polyubiquitination and adapter recruitment, phagophore LC3 binding, engulfment by the autophagosome, and lysosomal digestion [75,76] (Fig. 1).

### 2.2. PINK1/Parkin-independent mitophagy

**Fig. 2.**
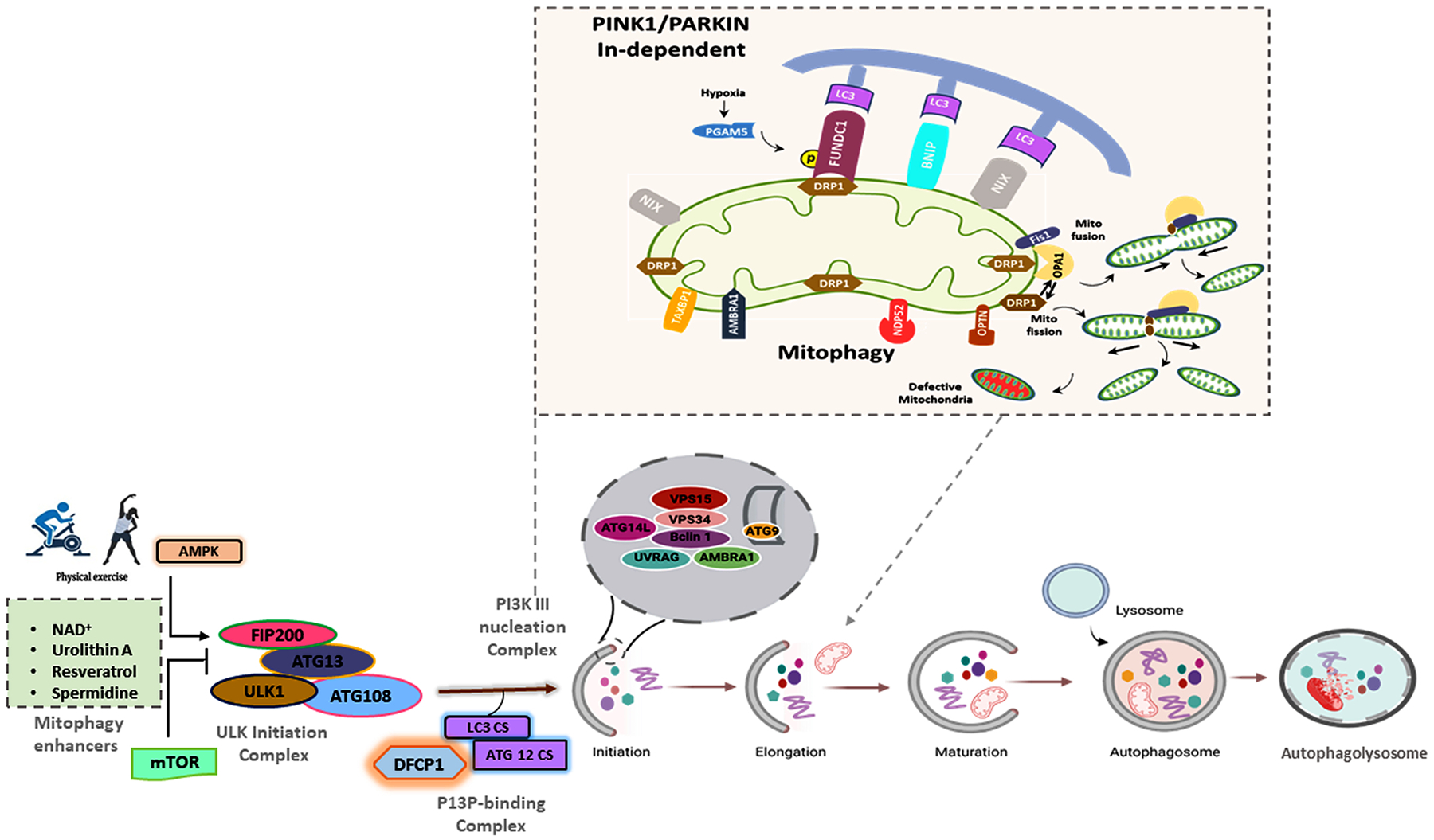
Mechanism of PINK1–Parkin-independent mitophagy. Specialized receptors, like NIX, BNIP3, and FUNDC1 are expressed on the OMM in response to stress stimuli. These receptors directly interact with LC3 to mediate mitochondrial elimination. In response to hypoxia, phosphorylation of NIX and BNIP3 enhances their associations with LC3. FUNDC1 also recruits LC3 in response to hypoxia, and FUNDC1 phosphorylation promotes fission of damaged mitochondria through the recruitment of DRP1 and Opa1 on the mitochondrial surface. Formation of a double-layered membrane phagophore within the cytosol requires the PI3KC3 complex. The complex then binds ATG resulting in LC3-II conjugation to phosphatidylethanolamine (PE) which drives autophagosomal membrane expansion. The membrane grows to enwrap a portion of the cytosol, forming an autophagosome. In the final step of the process, lysosomes fuse with the autophagosome, releasing lysosomal hydrolases into the interior, resulting in degradation of the vesicle contents, including mitochondria.

In response to hypoxic conditions PINK1/Parkin-independent mitophagy is initiated by the OMM proteins. Hypoxia leads to transcriptional upregulation of BNIP3 and Nix by the HIF 1/2 transcription factors, which are stabilized under hypoxic conditions. This leads to subsequent mitophagy events. Structurally, BNIP3 and Nix are single-pass transmembrane proteins targeted to the mitochondria, and the cytosolic tails contain LIR motifs. Phosphorylation at the Ser34 and 35 residues, and the Ser17 and 24 residues, flanking the LIR motifs in Nix and BNIP3 respectively, promote interactions with LC3 under stress conditions. LC3 can be recognized by mitochondrial receptor proteins including FUNDC1, BNIP, and NIX to promote the completion of the phagophore, thus targeting the mitochondrion for mitophagy [76]. NIX protein plays an important role in mitochondrial quality checks. NIX accumulation on the mitochondrial surface flags mitochondria for mitophagic degradation [74]. BNIP3 is also able to bind phagophore LC3 to initiate mitophagy through Opa1, in conjunction with Fis1 and Drp1 which initiate fission [75]. The FUNDC1 receptor also promotes mitochondrial clearance during hypoxic conditions; it interacts with the ER and recruits Drp1 during mitochondrial fragmentation. FUNDC1 is a good protein marker for stress-induced mitophagy [76] (Fig. 2).

### 2.3. Pharmacological modulation of mitophagy

Defective mitochondrial biogenesis and accumulation of dead mitochondria are the main phenomena observed in age-associated diseases such as AD. Newly developed pharmacological treatments and novel chemical modulators like urolithin A [77], tomatidine [78,79], and actinonin [80] might be used to promote the efficient removal of dead and/or damaged mitochondria and restore energy homeostasis. Pharmacologic agents and natural supplements that target mitophagy are potential drugs which may enhance the quality of mitochondria in aging individuals and ameliorate age-related diseases. To this end, several synthetic chemicals and natural compounds have been studied which help to regulate mitophagy and eliminate dysfunctional mitochondria.

### 2.4. Mitophagy enhancer NAD^+^

Nicotinamide adenine dinucleotide (NAD) and nicotinamide adenine dinucleotide phosphate (NADPH) are essential cofactors involved in several cellular redox reactions [81]. Nicotinamide adenine dinucleotide is a well-known coenzyme found in all living cells and is central to metabolism. NAD exists in two forms: the oxidized NAD^+^ form and the reduced NADH form [82]. NAD^+^ plays an important role in reductive reactions such as lipid biosynthesis and the synthesis and repair of DNA. The balance state of NAD is defined by the NAD^+^/NADH ratio, which is essential for the normal redox state of a cell. The decline of this ratio with age has been reported in multiple organs, including brain, liver, muscles, and adipose tissue [83].

NAD is involved in oxidation–reduction reactions critical for glycolysis, fatty acid oxidation, the TCA cycle, and, importantly, complex I of the mitochondrial respiratory chain which is a key regulator of autophagy. NAD^+^ protects against age-dependent metabolic impairment and promotes longevity through the NAD^+^ dependent enzymes like silent information regulators (sirtuins). Currently, seven sirtuins have been reported [84], each exhibiting distinct subcellular localization and performing specific functions.

**Fig. 3.**
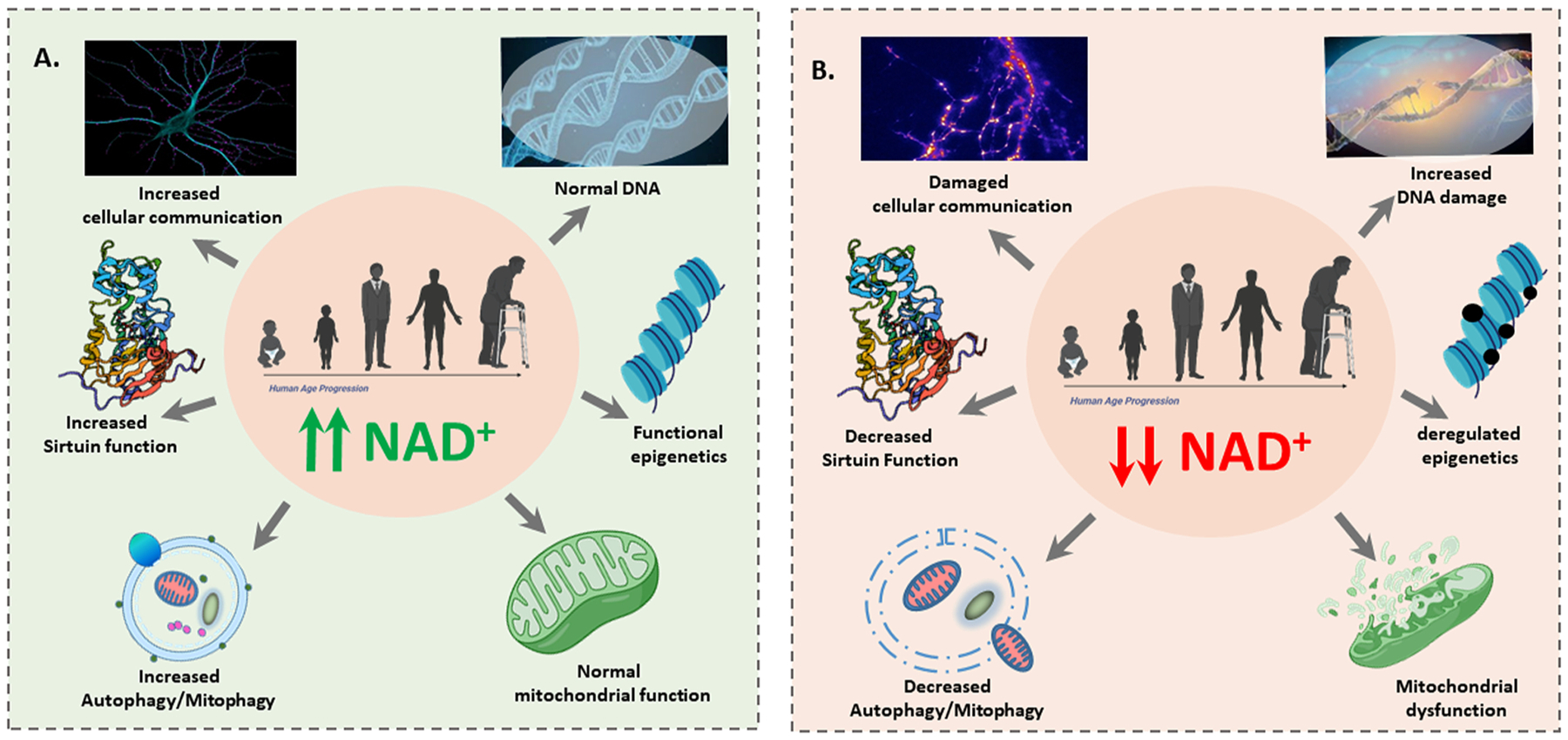
**A. Normal NAD^+^ levels are important for health**. A schematic representation of healthy NAD^+^ levels, which are required for several functions including repair of DNA, epigenetic regulation of gene expression, mitochondrial functions, effective mitophagy/autophagy, and Increased Sirtuin function. **B**. The decline of NAD^+^ is a core hallmark of Alzheimer’s disease and aging. A decline in NAD^+^ levels contributes to defects in DNA repair, deregulated epigenetics, mitochondrial dysfunction, deregulation of mitophagy/autophagy, and decreased Sirtuin function.

NAD^+^-dependent transcriptional regulation of sirtuins is important to the study of both NAD^+^ sensors and of NAD^+^ regulatory mediators. The enzyme-substrate mechanisms of NAD^+^-consuming proteins, such as sirtuins, poly (ADP-ribosyl) polymerases (PARPs), and cyclic ADP-ribose synthases (CD38 and CD157) have been well studied. These include NAD^+^ consuming sirtuins that regulate a broad range of cellular functions via NAD^+^-dependent deacetylation, and enzymes that generate bioactive second messengers including cyclic AMP, cyclic GMP, inositol triphosphate, diacylglycerol (Fig. 3). Sirtuins are directly linked to NAD^+^ signaling, and have shown profound consequences for lifespan and the development of age-associated diseases including AD. NAD^+^-dependent sirtuin activation of autophagic processes occurs when rate-dependent increases in the NAD^+^/NADH ratio activate SIRT1, which then activates autophagy by directly deacetylating ATG proteins.

NADPH eliminates cytoplasmic reactive oxygen species, which can also activate autophagy [85]. These events, in turn, activate SIRT1-linked transhydrogenase activity resulting in the interconversion of NADPH and NAD^+^ into NADP^+^ and NADH [86]. In the diseased state, this activity is inhibited by reactive oxygen species which accumulate following the loss of the glutathione peroxidase-glutathione reductase system [87].

Increased production of cytoplasmic reactive oxygen species in diseased/aged cells is caused by various pathological protein interactions with Aβ or P-tau, including the interaction of Aβ/P-tau with Drp1 and the interaction of Aβ/P-tau with VDAC1. Increased Drp1 in cells leads to defective mitophagy [117]. These effects lead to suppressed glutamate dehydrogenase expression and suppressed autophagy by mTORC1-mediated signaling. Pharmacological modulation of intra-cellular NAD^+^ via supplements and/or treatments stimulates a rise in the NAD^+^-SIRT1 level which has been shown to enhance mitophagy in models of neurodegenerative diseases.

### 2.5. Urolithins

**Fig. 4.**
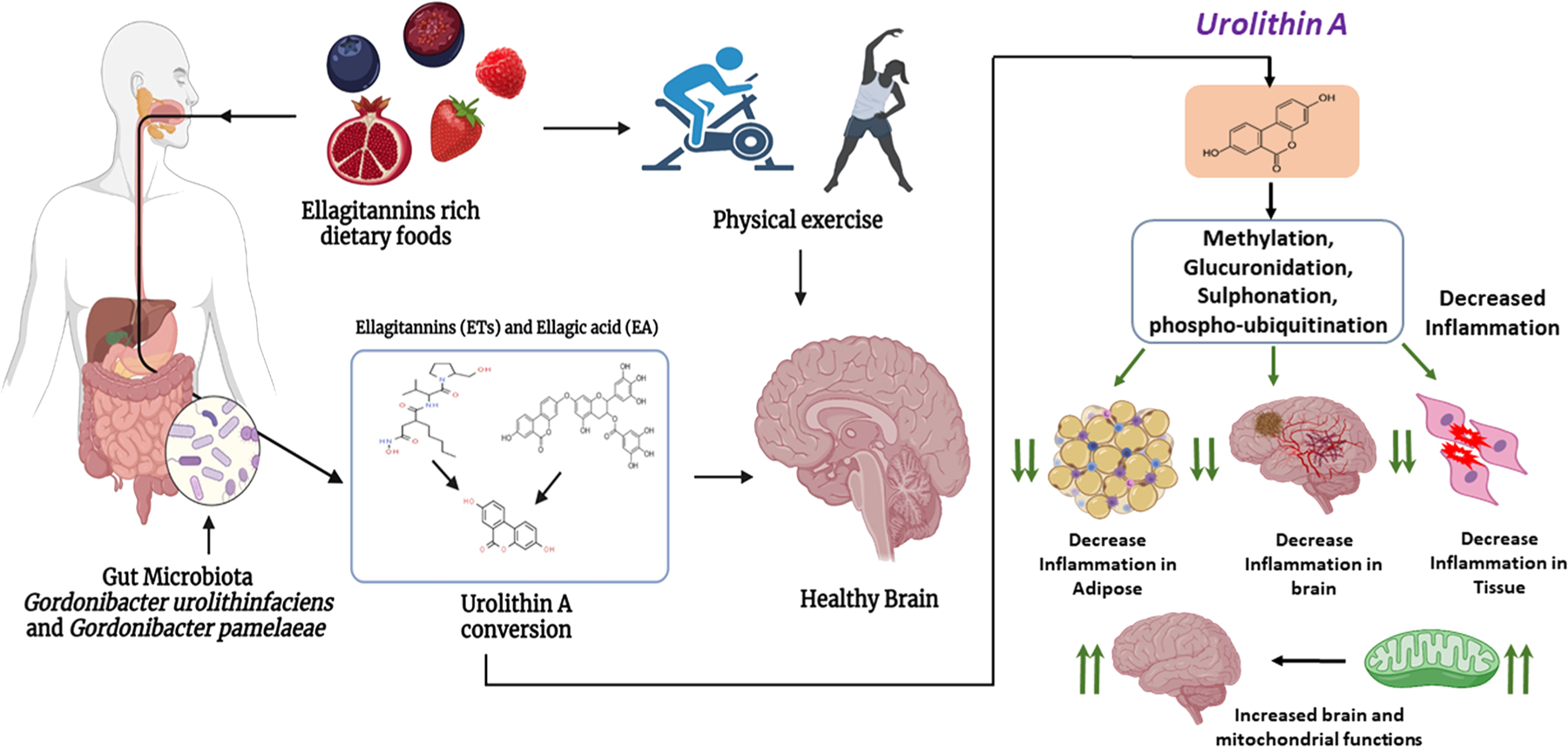
Several foods contain the natural polyphenols ellagitannins (ETs) and ellagic acid (EA). Ellagic-acid containing foods undergo gut microbial conversion to yield various forms of Urolithins. This conversion occurs only in the presence of microbes *Gordonibacter urolithinfaciens* and *Gordonibacter pamelaeae*. Urolithin supplementation improves mitochondrial and cellular health in age-related conditions, including AD. Urolithin A (UA) undergoes methylation, glucuronidation, and sulfation to yield conjugated forms of UA which decrease adipose, neuronal, and tissue inflammation. Decreased inflammation promotes healthy aging by improving mitochondrial function in the brain.

Urolithin is a microflora-derived metabolite produced by gut bacteria such as *Gordonibacter urolithinfaciens* and *Gordonibacter pamelaeae*, which is converted from ellagic acids (EA) and ellagitannins (ETs) into urolithin A [88]. Ellagitannins (ETs) and ellagic acid (EA) are complex polyphenols abundant in foods such as pomegranate, berries, and nuts (Fig. 4).

Urolithins belong to the class of organic compounds known as benzo-coumarins containing a 1-benzopyran moiety with a ketone group at the C2 carbon atom [89]. Hydrolysis of ellagitannins in the gut releases ellagic acid, which is further processed to urolithins by the loss of a lactone ring. Urolithins are absorbed into the intestine where they enter circulation. These circulatory urolithins are subjected to additional chemical transformations, including glucuronidation, methylation, and sulfation [90]. Urolithin formation depends on age, health status, dietary intake, and, importantly, the gut microbiome.

Glucuronidation occurs on Urolithin hydroxyl and amino groups in the presence of a glucuronyl transferase enzyme [91]. Glucuronidation helps in detoxifying foreign substances in humans and many animals. Methylation is involved in many biological functions including the regulation of gene expression, protein functions, and RNA processing. Methylated Urolithin A (UA) shows significant biological effects, including cell apoptosis, mitochondrial depolarization and down-regulation of Bcl-2/Bax [92]. Studies have shown that treatments of UA slow aging, reduce age-related diseases, and improve quality of mitochondria and overall health status [93]. UA prevents inflammation and promotes mitochondrial health, biogenesis and dynamics in different cell types and in different species including worms, mice, and humans [94–96].

Sulfated UA can in turn sulfate other cellular targets, improving multiple biological functions, such as the posttranslational modification of mitochondrial proteins, apoptosis, and mitophagy. The sulfate is transferred from UA to the tyrosine residues of target proteins by tyrosylprotein sulfotransferase enzymes of the Golgi apparatus. UA sulfation plays a role in protein-protein interactions, regulating G-protein-coupled receptors, coagulation factors, serine protease inhibitors, extracellular matrix proteins, and hormones. PINK1 and phospho-ubiquitin accumulation was observed in C2C12 mouse muscle myoblasts after treatment with UA [96], and increased levels of ubiquitinated and phospho-ubiquitinated mitochondrial proteins were observed following administration of UA to wild-type rodents, supporting the improvement of mitophagy by UA treatment [97].

Dietary consumption of EA-rich food has been demonstrated to suppress inflammatory cytokine release in the brains of AD mice [98]. Studies in rats reported that UA attenuates inflammatory IL-1β signaling by reducing the production of inflammatory mediators via the ERK, JNK, p38, and NF-kB pathways. Another study reported that UA induces mitophagy in vivo following oral consumption [98], and improvements in exercise capacity were observed in different models of age-related muscle atrophy [99]. UA supplementation studies in humans showed clear evidence that the functions of electron transport chain complexes I, II, and IV were increased, indicating improved mitochondrial health [100,101]. This finding was supported by evidence that dietary UA increased the mRNA and protein expression of autophagy/mitophagy and mitochondrial biogenesis genes [102]. Additional research on the beneficial effects of UA on mitochondrial function is emerging. UA supplementation or pharmacological treatments that target mitophagy pathways to improve mitochondrial health are proving to have beneficial effects in aging and age-related diseases, including AD and Parkinson’s disease (Fig. 4).

### 2.6. Resveratrol

**Fig. 5.**
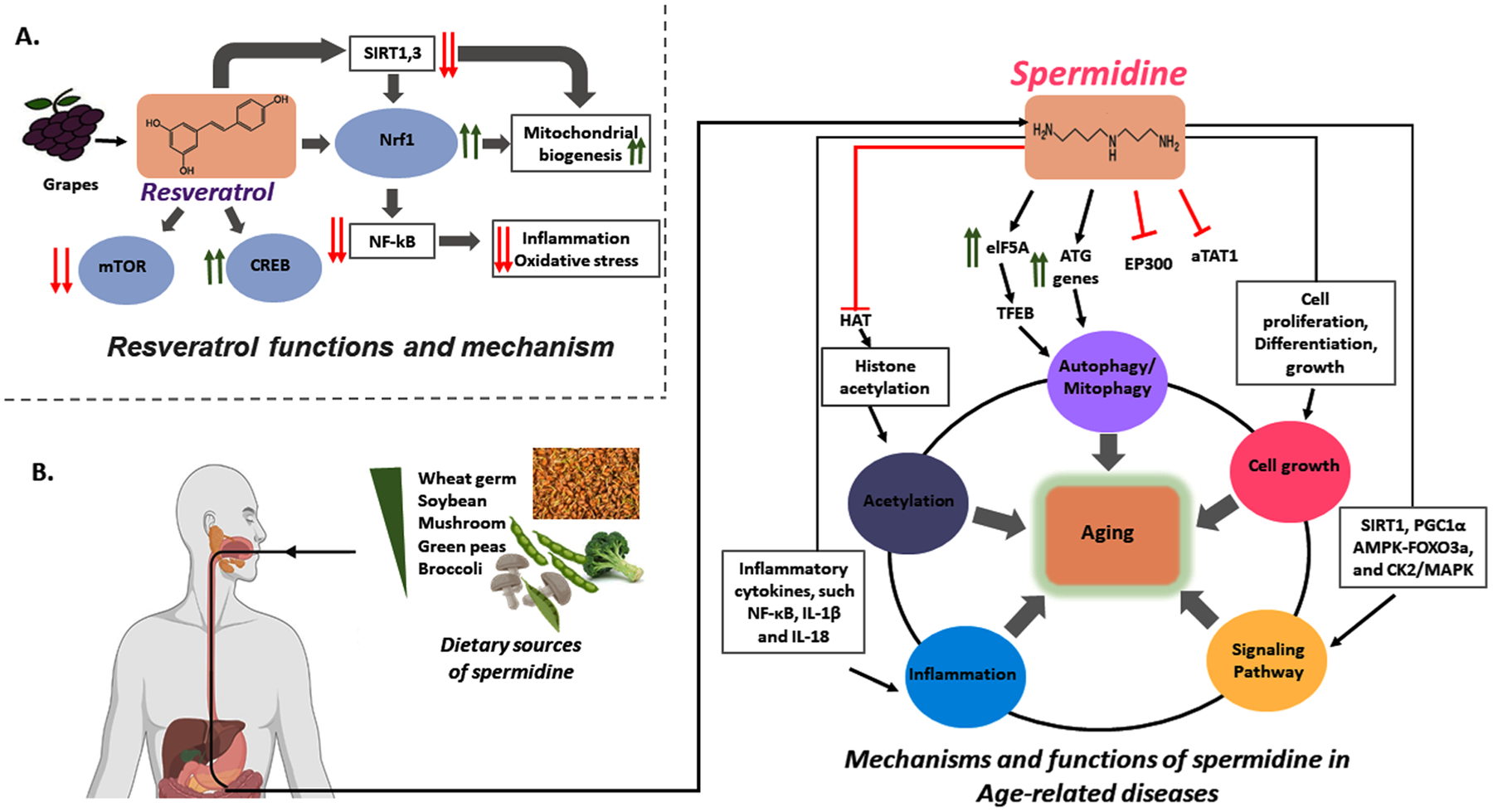
Protective effects of resveratrol. (**A**) Scheme of resveratrol’s actions to decrease inflammation and oxidative stress and to increase mitochondrial biogenesis by increasing SIRT1 and SIRT3 and by enhancing Nrf1 activation which inhibits Nf-kB signaling. Resveratrol counteracts mTOR signaling by activation of CREB, leading to improved mitochondrial biogenesis and healthy aging. (**B**) Several foods contain spermidine, which may improve mitochondrial function in AD and other age-related diseases. Spermidine induces autophagy by modulating the expressions of the ATG genes and the transcription factor elF5A. elF5A promotes the expression of the transcription factor TFEB which activates the expression of autophagy genes. Spermidine inhibits EP300, which directly promotes the acetylation of ATG proteins and indirectly stimulates deacetylation of tubulin due to inhibition of aTAT1. Spermidine exerts potent anti-inflammatory effects by suppressing multiple inflammatory cytokines, such as ROS, NF-κB, IL-1β and IL-18. Spermidine also suppresses histone acetylation. On the other hand, it can normalize signaling pathways affected by aging, such as SIRT1/PGC-1α, insulin/IGF, AMPK-FOXO3a, and CK2/MAPK.

Resveratrol is a naturally occurring phenol anti-aging agent, present in the skins of grapes, blueberries, raspberries, and mulberries. It exhibits numerous pharmacological properties including antitumor, antioxidant, antiviral, and neuroprotective effects in AD. Resveratrol reduces proinflammatory NF-kB signaling, restores normal expression of CREB protein in cells, and activates the Sirt1 pathway [103]. Activation of Sirt1 by resveratrol affects autophagy and mitophagy in both healthy and diseased states (Fig. 5A). Resveratrol also inhibits mitogen-activated protein kinases, and modulates the calcium-activated potassium channels, which are key for mitochondrial dynamics and biogenesis in aging and AD [104].

### 2.7. Spermidine

Polyamines are essential for cell proliferation and growth, and important for cellular metabolism [105]. Spermidine is a polyamine known to regulate various cellular processes, including DNA stability, cellular growth, differentiation, and apoptosis in aging and age-related diseases such as AD. Wheat germ, soybeans, mushrooms, rice bran, green peas and broccoli are rich sources of spermidine and arginine, which is a precursor of polyamines that increases the intestinal production of polyamines [106,107]. Studies in both animals [108] and humans [109] have reported that tissue concentrations of spermidine decline with age. Spermidine influences plasma membrane potentials via its effects on the Na^+^/K^+^-ATPase transporter and on Ca^2+^ influx by the glutamatergic N-methyl-D-aspartate receptor (NMDA receptor). Researchers have also found that spermidine helped to maintain the mitochondrial membrane potential and increase oxidative phosphorylation in isolated neuronal rat mitochondria [110]. Spermidine inhibits EP300, an acetyltransferase that inhibits the elongation of autophagosome membranes by acetylation of ATG proteins. This results in reduced ATG acetylation which induces autophagy, drawing further attention to the possible beneficial use of spermidine in AD and other age-related diseases [111].

Finally, EP300 has histone acetyltransferase activity, and thus has an epigenetic role as a transcriptional co-activator which is inhibited by spermidine. This finding, along with the finding that spermidine regulates the FOXO transcription factor, shows that spermidine may also promote mitophagy via epigenetic and transcriptional regulation in healthy and aged cells [112,113] (Fig. 5B).

### 2.8. Mitophagy enhancers with therapeutic applications

Recently, our lab investigated the protective effects of mitophagy enhancers against mutant APP and Aβ-induced mitochondrial and synaptic toxicities in cell models of AD [114]. We optimized doses of the mitophagy enhancers UA, actinonin, tomatidine, and nicotinamide riboside in immortalized mouse primary hippocampal (HT22) neurons. We transfected HT22 cells with a mutant APP overexpression plasmid, treated with the mitophagy enhancers, and assessed the mRNA and protein expression levels of mitochondrial dynamics, mitochondrial biogenesis, mitophagy and synaptic genes. We additionally assessed cell survival by MTT assay and mitochondrial respiration using a Seahorse Bioanalyzer in treated and control mAPP-HT22 cells. Finally, we assessed mitochondrial length and number in treated and control mAPP-HT22 cells using transmission electron microscopy [114].

In a second study, we evaluated the protective effects of mitophagy enhancers against P-tau-induced mitochondrial and synaptic toxicities in AD [115]. We transfected HT22 with a mutant tau expression plasmid and treated the cells with nicotinamide riboside, tomatidine, actinonin, and UA [115]. We assessed the mRNA and protein expression levels of mitochondrial dynamics, mitochondrial biogenesis, mitophagy and synaptic genes [115]. We also assessed cell survival, mitochondrial respiration, and mitochondrial morphology in treated and control mTau-HT22.

The results of the two studies were very similar. Relative to the control cells, the mAPP-HT22 and mTau-HT22 cells had increased expression of mitochondrial fission genes, decreased expression of mitochondrial fusion, synaptic and mitophagy genes, reduced cell viability, defective mitochondrial respiration, and excessively fragmented and smaller mitochondria. However, these events were reversed when the mAPP-HT22 and mTau-HT22 cells were treated with combinations of mitophagy enhancers. Cell survival was significantly increased, mRNA and protein levels of mitochondrial fusion, synaptic and mitophagy genes were increased, and mitochondrial fragmentation was reduced, resulting in mitochondria that were larger and fewer in number [114]. Of the treatments tested, UA showed the strongest protective effects against mutant APP- and Tau-induced mitochondrial and synaptic toxicities.

We then tested UA in combination with epigallocatechin gallate (EGCG), an abundant polyphenol in green tea which has been studied for its beneficial anti-neoplastic, anti-microbial and neuroprotective effects [116]. We found that the addition of EGCG to UA resulted in enhanced anti-AD effects relative to either single agent in the mTau-HT22 model [115]. These observations, together with findings from other research groups, indicate that mitophagy enhancers are promising as therapeutics for AD, and further studies are warranted.

Impaired mitophagy contributes to many pathological states, including synaptic dysfunction and cognitive deficits. Abnormal accumulation of Aβ and P-tau interacts with Drp1, causing excessive mitochondrial fragmentation and depletion of PINK1 and Parkin, leading to defective mitophagy in AD [117,118]. Pharmacological drugs and natural supplements like NAD^+^, urolithin, and resveratrol, along with daily exercise, can restore the functional aspects of mitophagy and help to eliminate diseased/aged mitochondria from neurons.

Evidence increasingly suggests that autophagy enhancing drugs like rapamycin, CCI-779, Glc, Glc-6-P, Torin1, perhexiline, niclosamide, and rottlerin attenuate mTORC1 signaling and enhance mitochondrial biogenesis in both diseased and healthy states [119]. Natural compounds like NAD^+^ [120], urolithins, and resveratrol have been shown to alter mitophagy via FOXO3a signaling, which potentiates parkin-PINK1-dependent mitophagy in diseased and stressed cells [121,122].

**Fig. 6.**
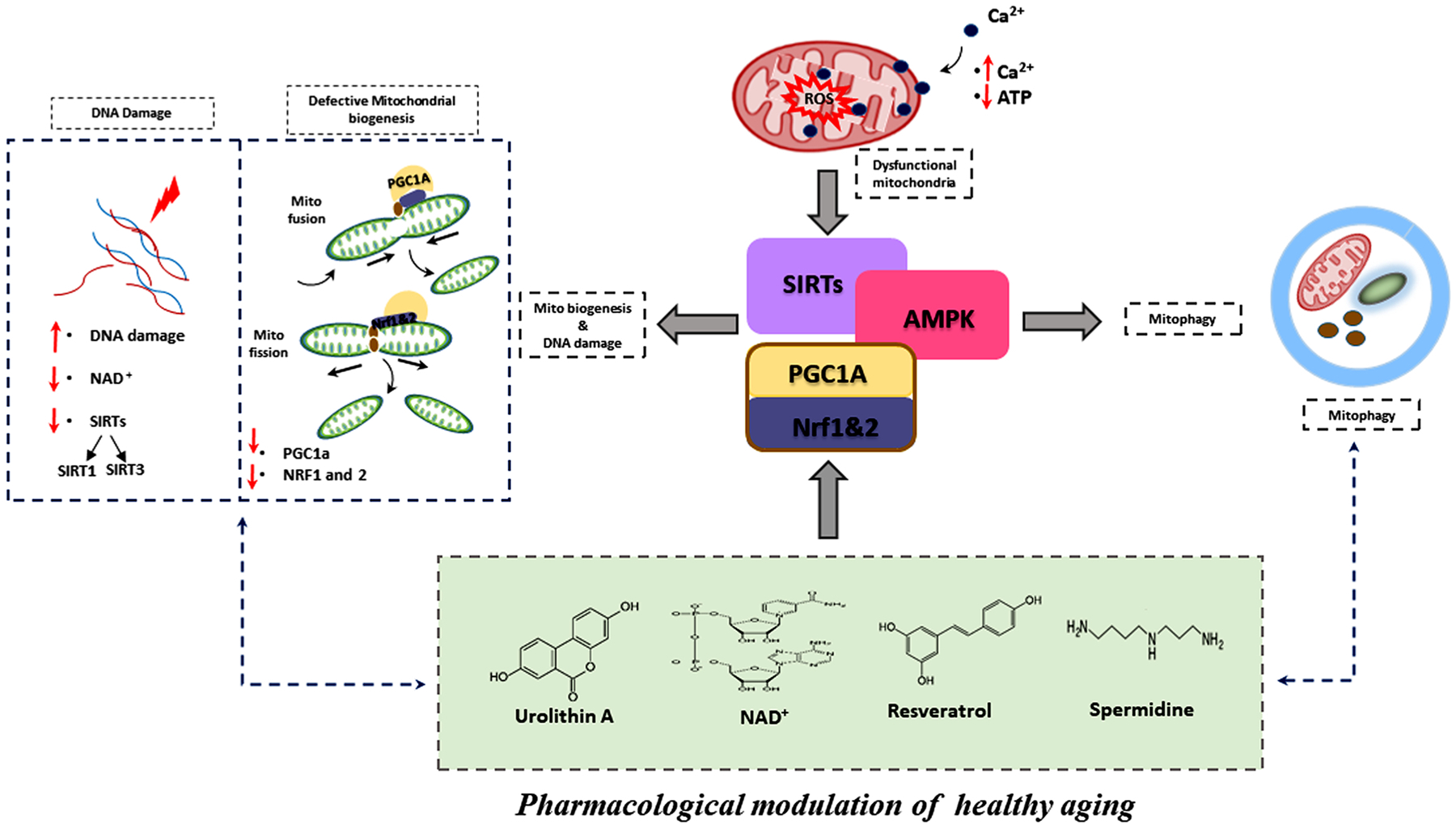
Pharmacological modulation of healthy aging with urolithin A, NAD^+^, resveratrol and spermidine. Mitochondrial dysfunction is characterized by elevated cytoplasmic calcium levels, ATP depletion, mitochondrial membrane potential failure, and increased reactive oxygen species production. Mitochondrial dysfunction is also associated with DNA damage and defective mitochondrial biogenesis. Mitophagy enhancers like Urolithin A, NAD^+^, Resveratrol and Spermidine, and physical exercise, promote effective mitophagy, mitochondrial dynamics, and mitochondrial biogenesis through the Sirtuin pathway. Pharmacological induction of PGC-1a through the AMPK-SIRT1 pathway stimulates mitochondrial biogenesis and improves mitochondrial health. PGC-1a and NRF2 activity affects mitochondrial detoxification and mitophagy induction. Pharmacological modulation of mitophagy and mitochondrial biogenesis may play an important role in mitochondrial homeostasis during stress, promoting healthy aging.

Resveratrol mitigates the effects of defective mitophagy by activating mitochondrial biogenesis via PGC-1α-SIRT1-AMPK signaling and restoring mitochondrial oxidative phosphorylation [123]. In addition, resveratrol inhibits the NF-kB pathway, reducing proinflammatory signaling. Another mitophagy enhancer, spermidine, was reported to attenuate mitochondrial depolarization by regulating the Ca^2+^ levels in mitochondria and, in turn, promote healthy mitochondrial biogenesis along with increased levels of PGC-1α, SIRT1, and TFAM [124,125]. Identification of compounds, validation of mitophagy enhancing effects, and characterization of the signaling and transcriptional pathways which mediate those effects will result in promising mitophagy enhancers that can be clinically evaluated as therapeutics to improve outcomes in aging and age-related diseases [126,127] (Fig. 6).

## 3. Conclusions and future directions

Mitochondrial abnormalities and synaptic damage are early events in the disease processes of AD. In AD, the accumulation of Aβ and P-tau plays a key role in mitochondrial dysfunction, synaptic damage, and neuronal loss. Dysfunctional mitochondria produce higher levels of reactive oxygen species, and the interactions of Aβ and P-tau with the mitochondrial proteins Drp1, VDAC, CypD, ABAD, PINK1, and Parkin enhance mitochondrial fragmentation and reduce mitophagy, leading to defective mitochondria in AD. Recent studies also suggest that mitophagy enhancers such as urolithin A, actinonin, tomatidine, and nicotinamide riboside are potential drug candidates that increase PINK1 and Parkin levels and increase the clearance of dead mitochondria [126,127].

Using immortalized mouse primary hippocampal (HT22) neurons transfected with mutant APP and mutant Tau expression plasmids, our lab recently investigated the protective effects of mitophagy enhancers against mutant Aβ- and mutant Tau-induced mitochondrial and synaptic toxicities in AD [114,115]. Mutant APP and mutant Tau-expressing HT22 cells showed increased expression of mitochondrial fission genes, decreased expression of mitochondrial fusion, synaptic and mitophagy genes, reduced cell survival, defective mitochondrial respiration, and excessively fragmented mitochondria of reduced lengths [114,115]. However, these events were reversed following treatment with mitophagy enhancers. In our work, UA showed the strongest protective effects against mutant APP and P-tau-induced mitochondrial and synaptic toxicities.

These and other observations [128,129] strongly suggest mitophagy enhancers are promising drugs to treat patients with AD and other tauopathies. However, these drugs need to be tested carefully using mouse models, particularly recently developed humanized Abeta knock-in (hAbKI) mice that express human amyloid beta peptide and exhibit late-onset AD features [130,131]. Currently, we are several mitophagy enhancers in late-onset hAbKI mice. Prior findings in other models support the safety and efficacy of this strategy; we expect that clinical trials will soon be warranted.

## Data Availability

No data was used for the research described in the article.

## Abbreviations:

AD: Alzheimer’s disease
ROS: Reactive Oxygen Species
mtDNA: mitochondrial DNA
Aβ: Amyloid beta
Drp1: Dynamin-1-like protein
ER: Endoplasmic reticulum
TEM: Transmission electron microscopy
Fis1: fission 1 protein
Miro: Mitochondrial Rho GTPase
Opa1: optic atrophy 1
Mfn1: Mitofusin-1
Mfn2: Mitofusin-2
Mid49: Mitochondrial dynamics protein MID49
Mid51: Mitochondrial dynamics protein MID51
Mff: Mitochondrial fission factor
NMDA: glutamatergic N-methyl-d-aspartate receptor
PGC-1α: Peroxisome proliferator-activated receptor gamma coactivator 1-alpha
NRF1: nuclear respiratory factor 1
NRF2: nuclear respiratory factor 2
TFAM: transcription factor A, mitochondrial
mAPP: Mutant amyloid beta precursor protein
PINK1: PTEN-induced kinase 1
BNIP3: BCL2/adenovirus E1B 19 kDa protein-interacting protein 3
LC3: Microtubule-associated protein 1A/1B-light chain 3
OPTN: Optineurin
FUNDC1: FUN14 domain containing 1
TBK1: TANK-binding kinase 1
SIAH1: E3 ubiquitin-protein ligase SIAH1
GSK3β: Glycogen synthase kinase 3 beta
CDK: Cyclin-dependent kinases
PKA: Protein kinase A
EF: helix-loop-helix structural domain
ERMCS: ER mitochondria encounter structures
LRRK2: leucine-rich repeat kinase 2
PARL: Proteases like presenilin-associated
OMA1: Metalloendopeptidase 1
AGEs: Advanced glycation end products
OMM: Outer membrane of mitochondria
ETC: Electron transport chain
SIRT1: Sirtuin 1
VDAC: Voltage-dependent anion channels
HT22: Mouse Hippocampal Neuronal Cell Line
mAPP: Mutant Amyloid Precursor Protein
P-tau: Phosphorylated Tau
